# Codon decoding by orthogonal tRNAs interrogates the *in vivo* preferences of unmodified adenosine in the wobble position

**DOI:** 10.3389/fgene.2024.1386299

**Published:** 2024-04-19

**Authors:** Margaret A. Schmitt, Jillyn M. Tittle, John D. Fisk

**Affiliations:** Department of Chemistry, University of Colorado Denver, Denver, CO, United States

**Keywords:** tRNA modification, inosine, orthogonal translation machinery, sense codon reassignment, wobble, translation, genetic code

## Abstract

The *in vivo* codon decoding preferences of tRNAs with an authentic adenosine residue at position 34 of the anticodon, the wobble position, are largely unexplored because very few unmodified A34 tRNA genes exist across the three domains of life. The expanded wobble rules suggest that unmodified adenosine pairs most strongly with uracil, modestly with cytosine, and weakly with guanosine and adenosine. Inosine, a modified adenosine, on the other hand, pairs strongly with both uracil and cytosine and to a lesser extent adenosine. Orthogonal pair directed sense codon reassignment experiments offer a tool with which to interrogate the translational activity of A34 tRNAs because the introduced tRNA can be engineered with any anticodon. Our fluorescence-based screen utilizes the absolute requirement of tyrosine at position 66 of superfolder GFP for autocatalytic fluorophore formation. The introduced orthogonal tRNA competes with the endogenous translation machinery to incorporate tyrosine in response to a codon typically assigned another meaning in the genetic code. We evaluated the codon reassignment efficiencies of 15 of the 16 possible orthogonal tRNAs with A34 anticodons. We examined the Sanger sequencing chromatograms for cDNAs from each of the reverse transcribed tRNAs for evidence of inosine modification. Despite several A34 tRNAs decoding closely-related C-ending codons, partial inosine modification was detected for only three species. These experiments employ a single tRNA body with a single attached amino acid to interrogate the behavior of different anticodons in the background of *in vivo E. coli* translation and greatly expand the set of experimental measurements of the *in vivo* function of A34 tRNAs in translation. For the most part, unmodified A34 tRNAs largely pair with only U3 codons as the original wobble rules suggest. In instances with GC pairs in the first two codon positions, unmodified A34 tRNAs decode the C- and G-ending codons as well as the expected U-ending codon. These observations support the “two-out-of-three” and “strong and weak” codon hypotheses.

## 1 Introduction

Organisms utilize essentially the same, universal genetic code to convert the information in their DNA genomes into functional proteins. With few exceptions, each of the 64 three nucleotide codons communicates the same signal to the protein translation machinery across the three domains of life. Through the course of evolution, alternative strategies to decode sense codons have developed (Knight et al., 2001). Organisms rely upon complements of tRNA molecules with disparate sets of anticodons and anticodon modifications in order to translate the same mRNA codons. Although the different tRNA complements translate a single mRNA sequence into a protein with a single primary sequence, the energetics of the tRNA/mRNA/ribosome interactions that are required to facilitate translation differ. Thus, the alternative sets of tRNA species lead to differing sets of “preferred” codons across organisms (Andersson and Kurland, 1990). Codon usage is, in part, related to the efficiency with which preferred codons are decoded (dos Reis et al., 2003). The major differences in decoding strategies follows the tripartite division of extant organisms into bacterial, archaeal, and eukaryotic domains (Woese and Fox, 1977).

One prominent difference between the strategies employed to read the genetic code relates to the manner by which the third (wobble) position of codons are decoded (Bjork and Hagervall, 2014; Agris et al., 2018). The wobble hypothesis postulated that some tRNA anticodons could decode multiple codons to explain how fewer than 61 tRNA species were able to translate the full suite of sense codons. The structure of the genetic code, together with the apparent activity of tRNA species, suggested that the first two bases of a codon were read in canonical Watson-Crick fashion and that some “wobble” was allowed in the third codon position (Crick, 1966). At the time when the wobble hypothesis was formulated, the genetic code had not yet been elucidated fully and information on tRNA sequences, modifications, and their interactions with codons was incomplete.

Inosine modification is the prototypical example of the way in which a modified anticodon base in a tRNA might be capable of decoding multiple codons. Inosine (abbreviated as “I”) is a modified adenosine base, produced by deamination of the exocyclic N6 amine. When the wobble hypothesis was presented, inosine modification had been found at position 34 in the anticodons of several tRNA species, the location that interacts with the third codon position (Ingram and Sjoquist, 1963; Holley et al., 1965; Sӧll et al., 1966). According to the original elaboration of the “wobble rules”, adenosine was expected to pair exclusively with uracil, while inosine was expected to pair with uracil, cytosine, and adenosine. Modern genomic surveys have shown that tRNAs encoded as A34 are nearly universally converted to inosine (Chan and Lowe, 2016). Unmodified A34 tRNAs are exceedingly rare.

The distribution and prevalence of inosine modification is divergent across the three domains of life (Ehrlich et al., 2021; Srinivasan et al., 2021). In archaea, genes for tRNAs with A34 anticodons, the precursor to I34 anticodons, are absent. In the bacterial domain, typically only a single tRNA gene with an A34 anticodon exists. This tRNA is modified to I34 and reads the arginine CGU, CGC, and CGA codons. A handful of A34 tRNAs, some of which are inosine modified, have recently been identified in other codon four boxes in a small number of bacterial species. Even with these findings, inosine usage remains highly restricted in the bacterial domain. Inosine modification is much more widely employed in eukaryotes, where each of the amino acids degenerately encoded by three, four or six codons, with the exception of glycine, typically utilize I34 tRNAs. The U-, C-, and A-ending codons in the TAPSLIVR family (where “TAPSLIVR” is the acronym containing the one letter amino acid codes for threonine, alanine, proline, serine, leucine, isoleucine, valine, and arginine) are all decoded by a major tRNA species with an I34 anticodon (Rafels-Ybern et al., 2015; Rafels-Ybern et al. 2018; Rafels-Ybern et al. 2019).

Inosine is introduced into tRNAs post-transcriptionally by adenine deaminase acting on tRNA (ADAT) enzymes (Gerber and Keller, 1999; Torres et al., 2014). Each inosine-modified tRNA is genomically encoded and initially transcribed as an A34 tRNA, followed by subsequent quantitative deamination to inosine. In bacteria, the ADAT enzyme encoded by the *tadA* gene is homodimeric and typically has only the single aforementioned arginine tRNA as its substrate (Wolf et al., 2002). In eukaryotes, inosine is introduced into the TAPSLIVR tRNAs post-transcriptionally by a single, heterodimeric ADAT enzyme that recognizes the multiple substrate tRNAs (Gerber and Keller, 1999; Torres et al., 2014).

Previously, we and others have explored the extent to which orthogonal tRNA/aminoacyl tRNA synthetase (aaRS) pairs can be employed to break the degeneracy of the genetic code in *E. coli* (Kwon et al., 2003; Bohlke and Budisa, 2014; Zeng et al., 2014; Biddle et al., 2015; Lee et al., 2015; Mukai et al., 2015; Ho et al., 2016; Kwon and Choi, 2016). Canonical amino acids that are encoded by more than a single codon are potential targets for reassignment since one (or more) of the sense codons can be left to direct incorporation of the natively-encoded amino acid and one (or more) of the codons can be given a reassigned meaning. Breaking the degeneracy of the genetic code requires the identification or engineering of pairs of tRNAs and the aaRSs that recognize them which are “orthogonal” to the complement of tRNA/aaRS pairs naturally employed by the target organism (Liu and Schultz, 2010; Acevedo-Rocha and Budisa, 2016; Melnikov and Sӧll, 2019). The anticodon of the orthogonal tRNA is engineered to decode a sense codon typically assigned another meaning in the genetic code. In order to expand translation beyond the set of canonical amino acids, the orthogonal aaRS must recognize and aminoacylate its cognate tRNA with a noncanonical amino acid (ncAA). Even partial decoding of a targeted sense codon by the supplied orthogonal machinery can lead to incorporation of the ncAA.

The first demonstration of breaking the degeneracy of the genetic code utilized an orthogonal yeast tRNA species with an AAA anticodon to reassign the UUU phenylalanine codon in *E. coli* (Kwon et al., 2003). In an expansion of the concept demonstrated in this work, we have mapped the extent to which anticodon variants of the orthogonal *Methanocaldococcus jannaschii* (*M. jannaschii*) tyrosine tRNA/aaRS pair can be used to break the degeneracy of the genetic code for 17 of the 18 multi-codon canonical amino acids in *E. coli* (Biddle et al., 2015; Schmitt et al., 2018; Schwark et al., 2020). The *M. jannaschii* tyrosine pair is one of the two orthogonal pairs most commonly used to introduce ncAAs into proteins in response to stop codons. Variants of the *M. jannaschii* aaRS have been engineered to aminoacylate >100 different ncAAs onto the cognate tRNA (Dumas et al., 2015). Our system for broad exploration of codon reassignment exploits the native function of the *M. jannaschii* orthogonal pair: incorporation of tyrosine. We developed a gain of function screen to quantify introduction of tyrosine by engineered variants of the *M. jannaschii* tRNA in response to non-tyrosine codons at a critical position in green fluorescent protein.

The effectiveness of a given orthogonal pair for reassigning the meaning of a particular sense codon depends on multiple factors, including the efficiency of the aaRS charging its anticodon-engineered cognate tRNA, the competition mounted by the complement of native tRNAs, and the energetics of the codon-anticodon interaction itself. *E. coli* wobble codons are promising locations at which to infiltrate and expand the genetic code because the majority are decoded by endogenous tRNAs via U3/G34 or G3/U34 interactions. The anticodons of the introduced orthogonal tRNAs are engineered to Watson-Crick base pair with the targeted codon and potentially take advantage of favorable codon/anticodon energetics at the wobble position to improve reassignment efficiency.

The extent to which a particular engineered tRNA is able to discriminate between the targeted codon and closely-related codons is an important consideration in choosing an orthogonal system to precisely reassign a given codon. tRNAs with A34 anticodons engineered to target the U3 wobble codons were expected to readily discriminate between the U-ending and non-targeted C-ending codons. Our initial evaluation of the reassignment efficiencies of four sense codons, including His CAU, by the *M. jannaschii* pair revealed unexpected, nearly equal reassignment of the CAU and CAC codons (Biddle et al., 2016). Through sequencing of reverse transcribed tRNA species, we showed that the orthogonal tRNA engineered to have an AUG anticodon was partially modified to inosine by *E. coli* TadA. Typically, “orthogonality” of introduced translation machinery has been interpreted as a lack of crosstalk between the orthogonal tRNAs and host aaRSs and between host tRNAs and the orthogonal aaRSs (Furter, 1998; Wang et al., 2001; Cervettini et al., 2020). Crosstalk between the orthogonal translation components, particularly the tRNA, and other host enzymes, e.g., tRNA modifying enzymes, has not been broadly evaluated. Given the prevalence of tRNA modifications and their myriad roles in the conversion of genotype to phenotype, modification of orthogonal tRNAs by endogenous enzymes is not surprising. Understanding this space of interactions may inform the combination of orthogonal pair and reassignable codon chosen as systems with increasingly expanded genetic codes are engineered (Mukai et al., 2017; Dunkelmann et al., 2021).

In a previous publication, we quantified the codon reassignment efficiency of *M. jannaschii* tRNAs with 14 of the 16 possible ANN anticodons in *E. coli* (Schmitt et al., 2018). In each case, we evaluated the system for reassignment of targeted and non-targeted codons. Some of the introduced orthogonal A34 tRNAs behave as postulated through the wobble hypothesis, decoding the U3 codon nearly exclusively. Another group of introduced orthogonal A34 tRNAs translate other codons within the box with substantial efficiency compared to the efficiency of reassignment of the targeted codon. Decoding of C-ending codons by A34 orthogonal tRNAs suggested that additional anticodons in the context of the *M. jannaschii* tRNA body may be substrates for *E. coli* TadA. Here, we analyze Sanger sequencing of the cDNA products of reverse transcribed *M. jannaschii* tRNA species with 15 A34 anticodons as well as present the reassignment preferences of *M. jannaschii* tRNA_ACG_ targeting Arg CGU and an expanded investigation of translation of NNA and NNG codons by orthogonal A34 tRNAs.

Detection of inosine modification via Sanger sequencing does not map onto the extent that engineered orthogonal tRNAs discriminate between the targeted U-ending codon and closely-related, non-U-ending codons; the majority of *M. jannaschii* tRNAs with A34 anticodons are not inosine modified. Experimental characterization of the decoding preferences of authentic A34 anticodons either *in vivo* or *in vitro* is sparse, in part because of the rarity of unmodified A34 tRNAs across the domains of life. This set of directed sense codon reassignment experiments utilizes a single tRNA body with a single attached amino acid to interrogate the behavior of authentic A34 anticodons in the background of *in vivo E*. *coli* translation.

## 2 Methods and materials

DNA vector composition and construction have been described previously (Biddle et al., 2015; Biddle et al., 2016; Schmitt et al., 2018; Schwark et al., 2020). Orthogonal translation machinery vectors utilized in this study differ only at the identity of the nucleotides that correspond to positions 34–36 of the tRNA gene. GFP reporter vectors differ only at the identity of the nucleotides that specify the codon corresponding to position 66 of the superfolding GFP variant. The superfolder GFP mRNA sequence was designed to minimize usage of U3 codons for amino acids read via wobble interactions in *E. coli*. The gain of function, fluorescence-based screen for quantifying codon reassignment has been described (Biddle et al., 2015). Brief summaries of vectors and the screen are provided as Supplementary Material. The Supplementary Material also includes details on cell strains, oligonucleotide primer sequences, numbers of biological replicates evaluated for each codon reassignment measurement, and additional sequencing traces of reverse transcribed tRNAs.

### 2.1 Reverse transcription of *M. jannaschii* tRNAs for Sanger sequencing

RNAse Away was used routinely throughout the process to keep the work area free of RNAses, and all consumables used were certified RNAse free. The complement of tRNA molecules was extracted from NEB 5-alpha cells harboring an orthogonal translation machinery vector. Briefly, 10^9^ cells were pelleted from overnight cultures by spinning sufficient volumes at 17,000 x*g* for 2 min at room temperature. Cell supernatant was removed via pipette. The cell pellets were frozen overnight at −20°C. Following thawing on ice, 200 μL B-PER lysis reagent (Thermo Scientific) was added to each pellet. Cell lysis proceeded at room temperature for 30 min.

A 1:1 volume of 0.3 M NaOAc, 10 mM EDTA, pH 4.5 was added to lower the pH of the solution. 450 μL of water-buffered phenol:chloroform (5:1 ratio) was added to the lysate and vortexed three times for 60 s, 60 s, and 30 s, with lysates held on ice for 60 s between each step. Layers were separated by centrifugation at 15,000 x*g* at 4°C for 15 min. The aqueous, RNA-containing layer was transferred to 2.5 volumes of 100% ethanol to precipitate the nucleic acids. Following a brief vortex, the solution was stored overnight at −20°C.

Nucleic acids were pelleted by centrifugation at 15,000 x*g* at 4°C for 30 min. The supernatant was aspirated, and the pellet was allowed to air dry. Nucleic acids were resuspended in 100 μL of 1x DNAseI buffer (Thermo Scientific). One unit of DNaseI was added, and incubation proceeded at 37°C for 20 min. DNAseI was inactivated and removed by phenol:chloroform extraction and ethanol precipitation as described above. RNA was resuspended in 20 μL of 0.3 M NaOAc, 10 mM EDTA, pH 4.5 and quantified using a BioTek Synergy H1 or Synergy Neo2S plate reader. Each RNA extraction yielded 12–20 μg of RNA.

Reverse transcription was carried out using SuperScript IV reverse transcriptase (Thermo Scientific) according to the manufacturer’s instructions and primer Mj-RevTrans (sequence in Supplementary Table S1). Duplicate reactions without enzyme were included as negative controls to ensure subsequent amplification resulted from tRNA molecules as opposed to carried-through DNA. cDNA products from reverse transcription were amplified with Taq polymerase (New England Biolabs) using primers cDNA-amp-fwd and cDNA-amp-rev (sequences in Supplementary Table S1). PCR products were cleaned up using the GeneJET PCR spin kit (Thermo Scientific) and analyzed by gel electrophoresis. Products were sequenced with primer cDNA-amp-fwd by Azenta (formerly Genewiz, LLC). Sanger sequencing .ab1 files were converted to .xml files using abi2xml.exe (freely available at https://github.com/eriksjolund/abi2xml) Figures were generated by importing data lines from the .xml files into Microsoft Excel.

## 3 Results

The fluorescence-based screen for sense codon reassignment exploits the absolute requirement of tyrosine at position 66 in superfolder GFP for fluorophore formation (Zacharias et al., 2006). Reporter vectors with another codon at that position are co-transformed with the *M. jannaschii* tyrosyl aminoacyl tRNA synthetase and a variant of its cognate tRNA. Both the engineered orthogonal tRNA and endogenous tRNAs compete to decode the evaluated codon. Incorporation of tyrosine by the orthogonal tRNA in response to the codon at position 66 in the GFP reporter leads to production of fluorescent protein. Incorporation of a different canonical amino acid by the endogenous translation machinery leads to production of non-fluorescent protein. The mean per cell fluorescence of the system under evaluation is bracketed between a 100% fluorescence reference system in which the codon at position 66 encodes tyrosine and a 0% fluorescence reference system in which the codon at position 66 encodes another amino acid. Stated reassignment efficiencies represent the mean and standard deviation of measurements of the number of biological replicates reported in Supplementary Table S2.

**FIGURE 1:**
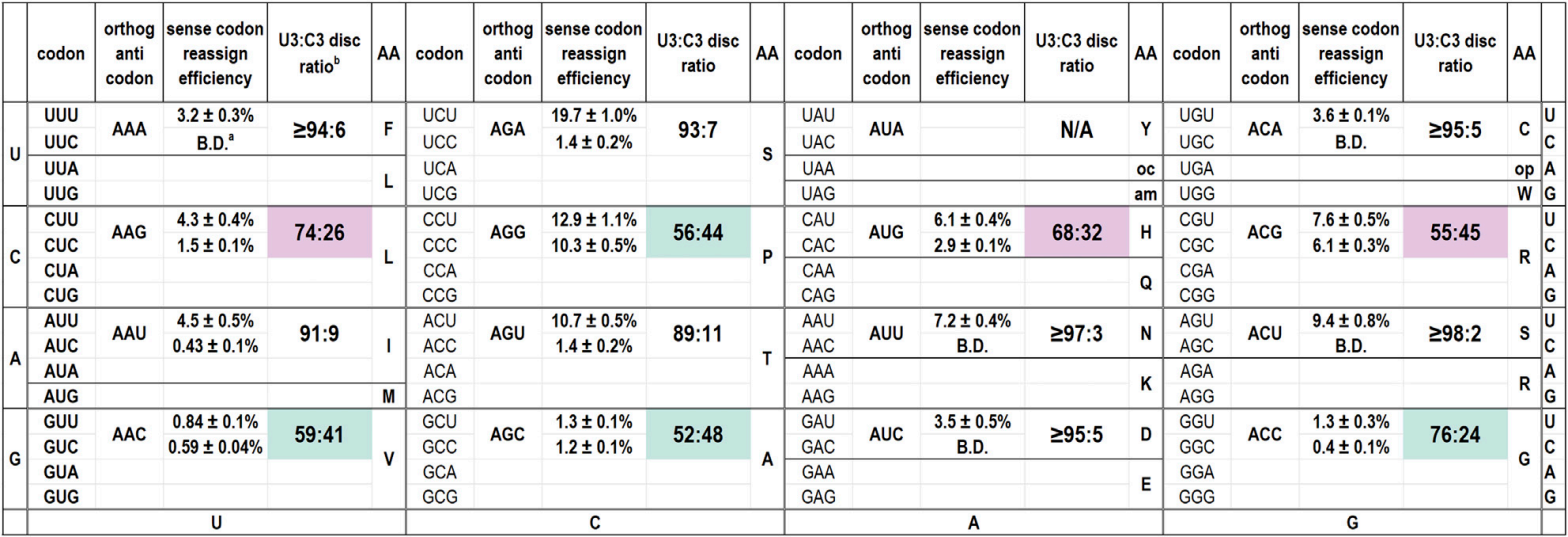
Codon reassignment efficiencies for U- and C-ending codons and U3:C3 discrimination ratios for 15 *M. jannaschii* orthogonal pair tRNA variants with ANN anticodons. ^a^ B.D. indicates that the reassignment efficiency of the particular codon/anticodon combination was below the limit of detection for the in cell assay (0.2%). The limit of detection is largely determined by the residual fluorescence of cells and growth media. ^b^ U3:C3 discrimination ratios are calculated as “[RE (reassignment efficiency) U-ending codon]/[RE U-ending codon + RE C-ending codon]”. For cases in which the C-ending codon was not reassigned above the limit of detection, the discrimination ratio is calculated as “[RE U-ending codon]/[RE U-ending codon +0.2]”. Shaded discrimination ratios indicate orthogonal tRNA anticodons with a notable tendency to decode the C-ending codon as well as the Watson-Crick base pairing U-ending codon. Pale pink shading indicates the three anticodons for which at least partial inosine modification was detected by Sanger sequencing of cDNA products from reverse transcribed tRNAs. Pale aqua shading indicates that no inosine modification was detected despite the tRNA discriminating between the U- and C-ending codons poorly.

We have evaluated the codon reassignment efficiencies of 15 of the 16 possible orthogonal tRNAs with ANN anticodons (Figure 1) (Schmitt et al., 2018). Our screen cannot be used to evaluate tyrosine codons. For tRNA_AUA_ that targets Tyr codons, decoding of the reporters by either the endogenous or orthogonal tRNAs produces a fluorescent protein. Of these tRNAs, six of 15 differ from the inosine-modified *E. coli* tRNA^Arg2^ at only one anticodon position, those with ANG or ACN anticodons. The eight remaining species differ from *E. coli* tRNA^Arg2^ at both position 35 and 36. Figure 1 is arranged to mirror the standard genetic code table and also presents the calculated U3:C3 discrimination ratios. Several of these tRNAs are capable of decoding both U- and C-ending codons.

**FIGURE 2:**
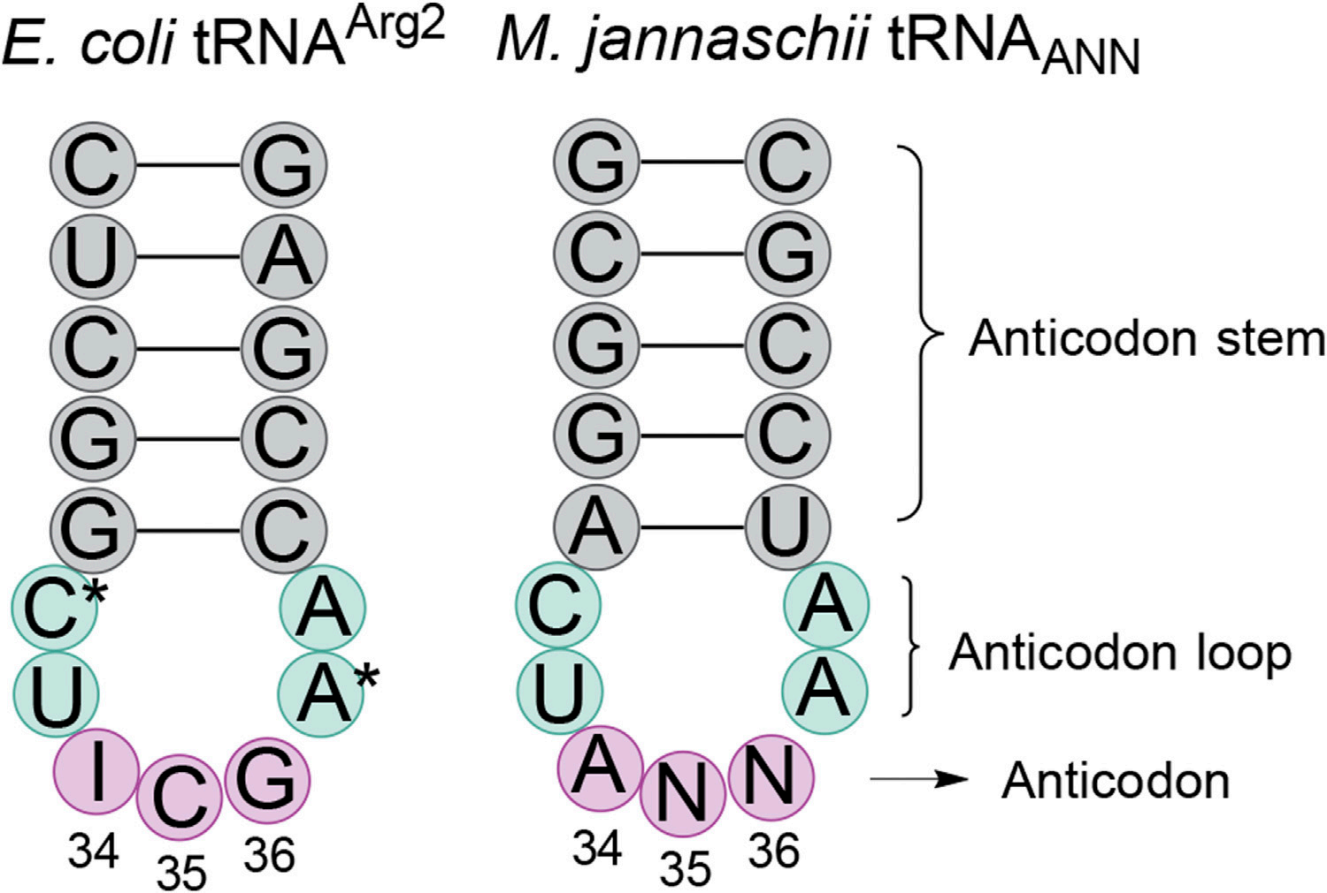
Comparison of the anticodon stem loop sequences of *E. coli* tRNA^Arg2^ and *M. jannaschii* tRNA^Tyr^_ANN_. The anticodon loop sequences (aqua shading) of the only *E. coli* tRNA transcribed as A34 and the commonly used *M. jannaschii* orthogonal tRNA are identical, save known nucleotide modifications in the *E. coli* tRNA. In tRNA^Arg2^, position 32 is modified to 2-thiocytidine and position 37 is modified to 2-methylthio-N6-isopentenyladenosine (ms2i6A). The anticodon stem (gray shading) is the same length in the two tRNAs. Depending on the anticodon (pink shading) of the orthogonal tRNA, the sequence may be 0, 1, or 2 nucleotides different than that of tRNA^Arg2^. The three anticodons that permit modification of A34 to inosine in the orthogonal tRNA are the only known modifications to the *M. jannaschii* tRNA when expressed in *E. coli*. Other modifications may exist.

After detecting partial inosine modification of the *M. jannaschii* tRNA_AUG_, we hypothesized that other orthogonal *M. jannaschii* tRNAs, specifically those capable of reassigning C-ending codons, might also be substrates for *E. coli* TadA adenine deaminase (Biddle et al., 2016). The anticodon loop sequences of *E. coli* tRNA^Arg2^ and the orthogonal *M. jannaschii* tRNA are identical, apart from known nucleotide modifications in the *E. coli* tRNA (Figure 2). While the identity elements for TadA recognition have not been definitively mapped, characterization of a limited set of minihelix substrates indicated that anticodon loop sequence was important for TadA activity. Structural evidence suggests that the sequence of the anticodon loop is exclusively involved in recognition (Elias and Huang, 2005; Kuratani et al., 2005; Losey et al., 2006).

**FIGURE 3:**
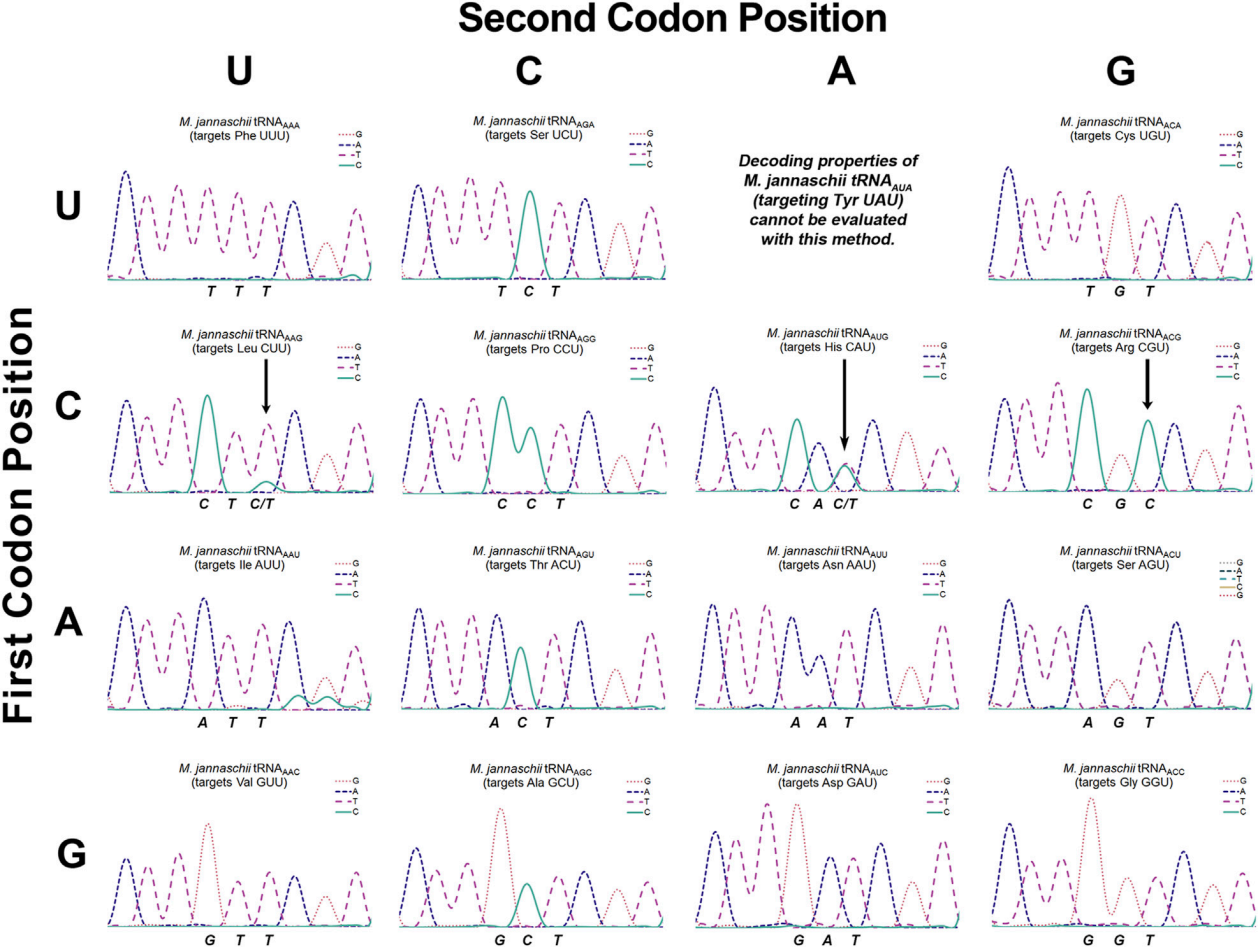
Sanger sequencing chromatogram traces of cDNAs reverse transcribed from isolated *M. jannaschii* tRNAs with ANN anticodons. The figure is arranged to mirror the standard genetic code table. Sequencing traces are placed according to the codon targeted for reassignment by the A34 tRNA. The sequenced strand corresponds to the reverse complement of the tRNA. The three nucleotides that specify the codon targeted for reassignment correspond to the anticodon of the tRNA and are stated below each trace. For example, the sequencing output for tRNA_AAA_ (targets Phe UUU) is TTT. Three arrows in traces for the CNU codon row indicate detectable inosine modification of the adenosine at position 34.

We have previously reported the targeted codon reassignment efficiencies for the majority of these orthogonal tRNAs (Schmitt et al., 2018). Here, we characterize the reassignment preferences of the 15th A34 tRNA, *M. jannaschii* tRNA_ACG_ and provide additional quantification of decoding non-U-ending codons by other A34 orthogonal tRNAs (Table 1). We report the observed extent of *in vivo* inosine modification, determined via sequencing of reverse transcribed cDNAs, for the 15 A34 tRNAs (Figure 3). We analyze the expanded suite of data in the context of recognition by *E. coli* TadA. In light of the absence of broad inosine modification, we describe the behavior of a wide set of tRNAs with authentic A34 bases in *E. coli* translation.

**TABLE 1:** Efficiency of codon reassignment to tyrosine by variants of the *M. jannaschii* orthogonal translation machinery with ANN anticodons.

tRNA anticodon	Codon targeted	Reassignment at PQU^a^ (%)	Reassignment at PQC (%)	Reassignment at PQA (%)	Reassignment at PQG (%)
AAA	Phe UUU	**3.2 ± 0.3**	B.D.^b^	-----^c^	-----
AAG	Leu CUU	**4.3 ± 0.4**	1.5 ± 0.1	B.D.	B.D.
AAU	Ile AUU	**4.5 ± 0.5**	0.43 ± 0.1	B.D.	0.27 ± 0.03
AAC	Val GUU	**0.84 ± 0.1**	0.59 ± 0.04	B.D.	0.35 ± 0.06
AGA	Ser UCU	**19.7 ± 1.0**	1.4 ± 0.2	B.D.	B.D.
AGG	Pro CCU	**12.9 ± 1.1**	10.3 ± 0.5	0.92 ± 0.1	1.0 ± 0.04
AGU	Thr ACU	**10.7 ± 0.5**	1.4 ± 0.2	B.D.	B.D.
AGC	Ala GCU	**1.3 ± 0.1**	1.2 ± 0.1	B.D.	B.D.
AUG	His CAU	**6.1 ± 0.4**	2.9 ± 0.1	B.D.	-----
AUU	Asn AAU	**7.2 ± 0.4**	B.D.	B.D.	B.D.
AUC	Asp GAU	**3.5 ± 0.5**	B.D.	B.D.	B.D.
ACA	Cys UGU	**3.6 ± 0.1**	B.D.	-----	-----
ACU	Arg CGU	**7.6 ± 0.5**	6.1 ± 0.3	0.91 ± 0.1	B.D.
ACU	Ser AGU	**9.4 ± 0.8**	B.D.	B.D.	0.36 ± 0.04
ACC	Gly GGU	**1.3 ± 0.3**	0.40 ± 0.1	B.D.	0.33 ± 0.01

Reassignment efficiencies in bold are those for the codon targeted for reassignment, which fully base pairs to the orthogonal tRNA via Watson-Crick interactions.

Reported reassignment efficiencies represent the mean and standard deviation of multiple biological replicates (typically >12) of each system. A complete list of the number of biological replicates that comprise each reported efficiency is provided in Supplementary Table S2.

^a^ For continuity, “P” and “Q” are used to represent the first two positions of the codon in the column headers. The sense codons evaluated with a single tRNA in each row are determined by substituting the complement of the nucleobases in the second and third anticodon positions for P and Q. The third codon position is specified in the column headers. For example, row 1 shows sense codon reassignment by the *M. jannaschii* tRNA_AAA_. Data in the PQU column are for reassignment at the UUU codon, with full Watson-Crick base pairing possible between codon and anticodon. Data in the PQC column are for reassignment at the UUC codon.

^b^ B.D. indicates that the codon was evaluated with the specified tRNA, and the measurement was below the detection limit of the in cell assay (0.2%).

^c^ “-----” indicates that the codon was not evaluated for reassignment by the specified tRNA.

The discrimination ratios can be thought of as “number of times out of 100 incorporation events a given tRNA selects the U-ending vs. C-ending codon”. Discrimination ratios were calculated by dividing the reassignment efficiency at the targeted, U-ending codon by the sum of the reassignment efficiencies for the U- and C-ending codons. For combinations of A34 tRNA/C3 codon reporters with efficiencies below the limit of detection (0.2%), the discrimination ratio is reported as “greater than or equal to [the quotient of reassignment of the U3 codon divided by reassignment of the U3 codon plus 0.2% (the limit of detection)”.

Discrimination ratios provide a normalized metric for how well individual orthogonal tRNAs decode U- and C-ending codons regardless of the absolute value of reassignment efficiencies observed. Orthogonal tRNA performance is dependent upon the effective concentration of the tRNA, which may change as aminoacylation efficiency by the aaRS changes in response to anticodon sequence, and the competition against the endogenous translation machinery. Discrimination ratios cancel the differences in aminoacylation level and competition with endogenous tRNAs inherent to each individual orthogonal tRNA and report how each single tRNA species decodes two codons, allowing normalized comparison across orthogonal tRNA species. For example, an orthogonal tRNA that decodes the U-ending codon with 50% efficiency and the C-ending codon with 5% efficiency has a discrimination ratio of 91:9. An orthogonal tRNA that decodes the U-ending codon with 4% efficiency and the C-ending codon with 0.4% efficiency also has a discrimination ratio of 91:9. Despite the disparate performance of each tRNA within the entirety of the endogenous translation machinery, both tRNA species incorporate their charged amino acid in response to a U-ending codon 91 times out of 100 orthogonal tRNA-directed incorporation events.

The degree to which U3:C3 codon discrimination may indicate inosine modification varies depending on the incorporation efficiency of the orthogonal tRNA and the detection limit of the assay. As targeted codon reassignment efficiency decreases, a higher percentage of the anticodon must be modified to inosine to reassign the C-ending codon above the limit of detection. Based on the strength of the I34/C3 pairing and expected weakness of an unmodified A34/C3 pairing, as little as 2% inosine modification of the tRNA molecules would be detected as “anomalous” reading of the C-ending codon by an A34 anticodon that reassigns the targeted U-ending codon at 20%. In the case of A34 anticodons that reassign their targeted codons with low efficiency (e.g., 1%), 40% inosine modification of the tRNA molecules would be required for detection as anomalous C-ending codon reassignment.

Our determination of the extent to which each of the tRNAs employed for sense codon reassignment were modified to inosine relied on Sanger sequencing of reverse transcribed tRNAs (Wolf et al., 2002). The tRNA substrates for reverse transcription were isolated using phenol:chloroform extraction. Reverse transcriptase recognizes inosine as guanine and triggers incorporation of cytosine. Unmodified adenosine, on the other hand, triggers incorporation of only thymidine (Wolf et al., 2002). The extent of inosine modification was determined by the ratio of T:C signal corresponding to position 34. Sequencing has been the standard method for detection of inosine modification; the method is semi-quantitative and reasonably sensitive. Other direct detection methods have been developed, but sequencing remains the most commonly used method of inosine detection (Vik et al., 2013; Torres et al., 2018; Knutson et al., 2020; Srinivasan et al., 2021). The observed baseline signals in the sequencing chromatograms suggest that ∼5% inosine modification would be clearly evident in chromatograms. Conclusions that individual tRNAs are not modified are based on sequencing traces where incorporation of cytosine, indicative of inosine modification, corresponding to position 34 is indistinguishable from baseline (Figure 3).

Figure 1 indicates the inosine modification status of each of the A34 tRNAs. Despite observation of a wide range of U3:C3 discrimination ratios, inosine modification is detected in only three A34 anticodons on the *M. jannaschii* tRNA body. Orthogonal tRNAs with three of four possible ANG anticodons: ACG (targeting Arg CGU), AUG (targeting His CAU), and AAG (targeting Leu CUU) showed indications of partial inosine modification. Figure 3 presents the relevant section of the chromatogram for Sanger sequencing the cDNA from reverse transcription of the 15 orthogonal tRNAs, arranged to mirror to the standard genetic code table. Only the chromatogram from sequencing *M. jannaschii* tRNA_AUG_ has been reported previously.

In some cases, the reassignment efficiencies reported in Figure 1 and Table 1 differ slightly from those previously reported. As additional biological replicates are added for any given system, we update the stated reassignment efficiency to reflect the accumulated data at the time of publication. Data gathered from multiple experiments across multiple days are readily comparable, demonstrating the robustness and stability of the screen. The consistency of observed reassignment efficiencies across experiments (e.g., batch-to-batch media preparation, person performing the experiment, fluorescence plate reader employed) is evident in the small standard deviations of the measurements.

### 3.1 Decoding preferences of orthogonal tRNAs with ANG anticodons

The family of A34 tRNAs with ANG anticodon sequences differ from the *E. coli* tRNA^Arg2^_ACG_ anticodon sequence at position 35, which base pairs to the second position of the mRNA codon. The engineered ANG anticodons target the CNU codons found in the same row of the genetic code table as the arginine four box (CGN codons). tRNA_AAG_ targets the Leu CUU codon, tRNA_AGG_ targets the Pro CCU codon, and tRNA_AUG_ targets the His CAU codon. Orthogonal tRNA_ACG_ targets the Arg CGU codon and, on the *M. jannaschii* tRNA body, has a transcribed anticodon loop sequence identical to that of *E. coli* tRNA^Arg2^ (Figure 2) (Chin et al., 2002). The *E. coli* tRNA^Arg2^ is modified at positions 32 and 37 in the anticodon loop. The modification state of the orthogonal *M. jannaschii* tRNAs has not been characterized beyond the evaluation of inosine modification at position 34.

The *M. jannaschii* tRNA_ACG_ is the only A34 tRNA variant for which we have not previously reported decoding efficiencies for the U- and C-ending codons. This orthogonal tRNA competes against the most abundant *E. coli* tRNA, and the CGU and CGC codons are not efficiently reassigned at 7.6% ± 0.5% and 6.1% ± 0.3%, respectively. The U3:C3 discrimination ratio for the *M. jannaschii* tRNA_ACG_ is 55:45. Sanger sequencing of the cDNA of the reverse transcribed orthogonal tRNA showed only C incorporated opposite position 34 of the anticodon, indicating a high level of inosine modification. The degree of modification evident in Sanger sequencing of the orthogonal tRNA_ACG_ was indistinguishable from the extent of modification determined from sequencing reverse transcribed *E. coli* tRNA^Arg2^, which is quantitatively modified.

As reported previously, a nearly 1:1 mixture of C and T is observed upon sequencing the reverse transcript of the *M. jannaschii* tRNA_AUG_ (Biddle et al., 2016). The 68:32 U3:C3 discrimination ratio for this tRNA suggests that the unmodified A34 version of the tRNA is selective for reading the U-ending codon, while the inosine-modified form is largely responsible for reading the C-ending codon. We identified several anticodon loop sequences that completely abrogate inosine modification from a small library of position 32, 37, and 38 variants. tRNA_AUG_ anticodon loop variants that authentically contain unmodified adenosine at position 34 display U3:C3 discrimination ratios greater than 95:5.

*M. jannaschii* tRNA_AAG_ targets the Leu CUU codon and displays a U3:C3 discrimination ratio of 76:24. Sanger sequencing of the cDNA showed a mixture of nucleotides corresponding to the A34 position, primarily T with a small amount of C, indicating partial modification to inosine (Figure 3). Relative peak areas suggest that approximately 15% of the anticodons are inosine modified. This degree of inosine modification coupled with the U3:C3 discrimination ratio suggests that the unmodified A34 tRNA modestly discriminates between U- and C- ending codons, in contrast to what is observed for histidine. Inosine modification was not detected by sequencing the *M. jannaschii* tRNA_AAG_ with an A37G mutation. Discrimination by *M. jannaschii* tRNA_AAG-A37G_ improved to 87:13 (Supplementary Figure S1).

The U3:C3 discrimination ratio for tRNA_AGG_ targeting Pro CCU, 56:44, is nearly identical to that of tRNA_ACG_ (arginine). Given the trends observed for other ANG anticodon tRNAs, we expected to see evidence of inosine modification in *M. jannaschii* tRNA_AGG_. Much to our surprise, no inosine modification was detected via Sanger sequencing of several cDNA preparations of reverse transcribed tRNAs (Figure 3 and additional traces in Supplementary Figure S2). The nearly-equal reassignment efficiency of the Pro CCU and CCC codons is the result of a mechanism(s) other than inosine modification. The apparent lack of discrimination between CCU and CCC codons in *Salmonella* by a mutant proline tRNA with an A34 anticodon, also without inosine modification, has been previously reported (Chen et al., 2002).

### 3.2 Decoding preferences of orthogonal tRNAs with ACN anticodons

The ACN anticodon family of tRNAs differ from the arginine sequence at position 36, which base pairs with the first position of the mRNA codon. The ACN anticodons target the NGU codons found in the same column of the genetic code table as the arginine four box (CGN codons). tRNA_ACA_ targets the Cys UGU codon, tRNA_ACU_ targets the Ser AGU codon, and tRNA_ACC_ targets the Gly GGU codon. For both tRNA_ACA_ and tRNA_ACU_, reassignment of the corresponding C-ending codon is below the limit of detection for our in cell assay. The calculated U3:C3 discrimination ratios are at least 95:5, depending on the efficiency of decoding the targeted U-ending codon (Figure 1). In both cases, only T is incorporated in response to the nucleotide corresponding to A34 in the Sanger sequencing of the reverse transcribed tRNAs (Figure 3). The U3:C3 codon discrimination ratios do not suggest partial inosine modification, and none is observed. In contrast, fluorescent GFP is produced at a level above that of the limit of detection when the *M. jannaschii* tRNA_ACC_ is evaluated for reassignment of the Gly GGC codon, leading to a calculated U3:C3 discrimination ratio of 76:24. This discrimination ratio is similar to that of the *M. jannaschii* tRNA_AAG_ (Leu CUU), which also differs from the ACG anticodon by a single nucleotide and for which partial inosine modification was detected. The chromatogram from Sanger sequencing of the *M. jannaschii* tRNA_ACC_ does not indicate inosine modification (Figure 3).

### 3.3 Codon discrimination preferences of A34 tRNAs differing at both the first and second codon positions

The remaining nine tRNAs with ADH anticodons differ from the arginine ACG anticodon at both positions 35 and 36 (The degenerate nucleotide D indicates “not C”, and the degenerate nucleotide H indicates “not G.”) We hypothesized that these tRNAs would be the least likely substrates for *E. coli* TadA. Indeed, sequencing chromatograms for the eight tRNAs we evaluated shows only T incorporated in response to the nucleotide corresponding to position 34, indicating an absence of inosine modification. The U3:C3 discrimination ratios for this group of tRNAs suggest a strong bias for U3 codons (Figure 1). Six of the eight tRNAs have U3:C3 codon discrimination ratios greater than or equal to 10 to 1.

Both the *M. jannaschii* tRNA_AAC_ (Val GUU) and *M. jannaschii* tRNA_AGC_ (Ala GCU) decode the corresponding U- and C-ending codons with nearly equal efficiency. Despite neither orthogonal tRNA reassigning the targeted U-ending codon efficiently, both around 1%, reassignment of the C-ending codon is well above the limit of detection. The U3:C3 discrimination ratios are 59:41 and 52:48, respectively.

*M. jannaschii* tRNA_AGA_ (Ser UCU), *M. jannaschii* tRNA_AGU_ (Thr ACU), and *M. jannaschii* tRNA_AAU_ (Ile AUU) also decode their corresponding C-ending codons above the limit of detection. However, given the efficiency with which each of these tRNAs decodes their targeted U-ending codon, U3:C3 discrimination ratios remain quite good, greater than or equal to approximately 10:1. The remaining three *M. jannaschii* A34 tRNAs (tRNA_AAA_ targeting Phe UUU; tRNA_AUU_ targeting Asn AAU; tRNA_AUC_ targeting Asp GAU) do not reassign the corresponding C-ending codon above the limit of detection. In each case, the tRNA reassigns the targeted U-ending codon with greater efficiency than either tRNA_AAC_ (Val GUU) or tRNA_AGC_ (Ala GCU), suggesting a strong bias for decoding U-ending codons within this group of tRNAs.

## 4 Discussion

### 4.1 Insight into the specificity of *E. coli* TadA

The precise determinants of adenosine deaminase TadA specificity have not been mapped, in part because bacteria typically only encode a single tRNA with a modifiable A34 anticodon. The original report describing the function of TadA as a tRNA deaminase relied on a small set of tRNA minisubstrates to evaluate the residues important for tRNA recognition (Wolf et al., 2002). Evaluation of 10 minihelix substrates suggested that the anticodon sequence was the major determinant of TadA recognition. The determinants of eukaryotic ADAT enzymes have been more fully elucidated (Frigolé et al., 2019). Our experiments employ a full tRNA body which presents its anticodon in an effective orientation for recognition by *E. coli* TadA, given that when the anticodon is AGC presumably full inosine modification is detected. With the exception of possible differences in modification state at positions 32 and 37, the anticodon loop of *M. jannaschii* tRNA_ACG_ is identical to that of *E. coli* tRNA^Arg2^ (Bjork and Hagervall, 2014). We examined 15 of 16 possible A34 anticodon variants; the extent of inosine modification of these tRNAs should more precisely map the anticodon elements important for TadA recognition.

cDNA sequencing revealed indications of inosine modification in only three out of 15 tRNAs evaluated. Just two of the three modified tRNA species are highly modified. Sequencing suggests that the TadA deaminase enzyme is very specific for the sequence of the *E. coli* tRNA^Arg2^ anticodon and anticodon loop. For both the AAG (Leu) and AUG (His) anticodons, each mutation to positions 32, 37 and/or 38 that was evaluated was sufficient to block recognition by TadA. Our results are consistent with studies employing minihelix substrates and recent evaluations of inosine tRNA modification in *Oenococcus oeni*, a bacterial strain that encodes four tRNAs with A34 anticodons, only two of which are inosine modified (Wolf et al., 2002; Rafels-Ybern et al., 2018; Rafels-Ybern et al., 2019).

### 4.2 Extent of A3 and G3 reading by A34 orthogonal tRNAs

The expanded wobble rules suggest that unmodified A34 tRNAs are capable of decoding any nucleotide at position three of the codon (Bjork and Hagervall, 2014; Agris et al., 2018). As we considered the combination of U3:C3 discrimination ratios and detectable inosine modification, we expanded our set of evaluated codons and measured the decoding properties of *M. jannaschii* tRNA_ANN_ species to the corresponding A- and G-ending codons, regardless of whether the codons were synonymous with the U- and C-ending codons. Table 1 presents a nearly complete evaluation of the reassignment efficiencies of *M. jannaschii* A34 tRNAs within their respective codon boxes. Measurements shaded in pale pink have not been previously reported.

Across the 12 A34 tRNAs which have been fully evaluated, six tRNAs show no reassignment of either the A- or G-ending codons. A single tRNA, *M. jannaschii* tRNA_AGG_ (Pro CCU), decodes all four codons above the limit of detection. This observation adds to the conclusion that factors other than inosine modification are influencing the behavior of this tRNA in *E. coli* translation. The CCA and CCG codons are each reassigned approximately 1% of the time. A single tRNA, *M. jannaschii* tRNA_ACG_ (Arg CGU), reassigns the CGA codon above the limit of detection (0.91% ± 0.1%) and does not reassign the CGG codon. This observation is unsurprising given the decoding preferences of tRNAs with inosine at position 34.

tRNA_ACU_ (Ser AGU) represents the only instance of an *M. jannaschii* A34 tRNA decoding only the U- and G-ending codons. Reassignment of the C- and A-ending codons is below the limit of detection. The AGG codon is the least commonly used sense codon in *E. coli* and is decoded by the least abundant endogenous tRNA species (Dong et al., 1996). The AGA codon is slightly more frequently used in *E. coli*, and an additional endogenous tRNA is utilized to decode it. This favorable competition environment to decode AGG, relative to even AGA, may explain the quantified reassignment preferences of the introduced tRNA_ACU_ and is one reason this codon has been explored for genetic code expansion (Zeng et al., 2014; Lee et al., 2015; Mukai et al., 2015; Biddle et al., 2022).

The remaining three tRNAs, tRNA_AAU_ (Ile AUU), tRNA_AAC_ (Val GUU), and tRNA_ACC_ (Gly GGU), decode both the C- and G-ending codons above the limit of detection and not the A-ending codon. The relative order of codon preference observed in an *in vitro* study was that A34 tRNAs generally preferred U > C > G > A (Boren et al., 1993).

### 4.3 Function of unmodified A34 tRNAs in translation

We observed a wide range of U3:C3 discrimination ratios; sequencing indicated inosine modification in only three out of 15 tRNAs evaluated. Our data describe a wider examination of the function of authentic A34 tRNAs than has been previously performed. The measurements of A34 tRNA decoding specificity speak to two related and relatively unstudied questions. 1. How do A34 tRNAs function in translation (Chen et al., 2002)? 2. Why are unmodified A34 tRNAs essentially absent from the tRNA complements of extant organisms (Ehrlich et al., 2021)? The wide range of observed U3:C3 discrimination ratios suggests that the functions of adenosine wobble interactions in codon recognition are idiosyncratic. Significant evidence that the effects of individual tRNA modifications also seem to be tRNA and codon context dependent exists (Phizicky and Alfonzo, 2010).

The set of directed sense codon reassignment experiments by *M. jannaschii* tRNA_ANN_ variants employ a single tRNA body with a single attached amino acid to interrogate the behavior of different anticodons in the background of *in vivo E*. *coli* translation. The major variables in our evaluations are the identity of the codon/anticodon pair measured and the subset of *E. coli* tRNAs against which the orthogonal tRNA competes. Our results greatly expand the set of experimental measurements of the function of A34 tRNAs in *in vivo* translation systems (Boren et al., 1993; Inagaki et al., 1995; Takai et al., 1999; Chen et al., 2002; Agris et al., 2018). The pattern of discrimination appears to be consistent with the “two-out-of-three” and “strong and weak” codon decoding hypotheses (Lagerkvist, 1978; Grosjean and Westhof, 2016).

Reading of codons by A34 tRNAs was addressed only in passing in Crick’s original discussion of the wobble hypothesis. Unmodified A34 anticodons were expected to read only U-ending codons (Crick, 1966). A major component of the argument supporting the wobble hypothesis was that modification of A34 tRNAs to inosine expanded the set of codons read by a single tRNA and thus reduced the set of tRNAs needed to read the genetic code. Within the anticodon sequences available at the time, inosine was present in several tRNA species. Those tRNAs were capable of pairing with U-, C-, and A-ending codons. As more tRNA sequences were determined, the facts that unmodified A34 tRNAs were extremely rare and that eukaryotes, in particular, largely utilized I34 tRNAs to read three and four codon boxes became increasingly clear.

While broadly correct in outline, the wobble hypothesis has been updated. The first adjustments reconciled decoding mechanisms in *Mycoplasma* and mitochondria, both of which utilize highly abbreviated tRNA complements. Identification of the large number of modified tRNA nucleotides in the anticodon stem loop and appreciation for their impact on codon/anticodon pairing has further refined the original hypothesis (Agris, 1991; Yokoyama and Nishimura, 1995; Grosjean and Westhof, 2016; Agris et al., 2018).

Unmodified A34 tRNAs are extremely rare, and their decoding properties have only been studied in a few instances (Rafels-Ybern et al., 2018; Rafels-Ybern et al., 2019). The majority of tRNAs that are encoded with an A34 anticodon are modified to inosine. In eukaryotes, each of the TAPSLIVR amino acids are primarily introduced by tRNAs with inosine at position 34 (Rafels-Ybern et al., 2018; Rafels-Ybern et al., 2019). In bacteria, only a single tRNA with an inosine-modified A34 is commonly employed to introduce arginine in the CGN codon box. Archaea do not typically utilize any A34 tRNAs (Phillips and de Crécy-Lagard, 2011). A recent analysis of the encoded tRNA sets from over 1,000 bacterial and eukaryotic genomes representing all major phylogenetic taxa confirmed that A34 tRNAs are extremely rare in bacteria and archaea. A handful of instances of bacterial genomes encoding additional A34 tRNAs beyond an arginine-incorporating variant are evident (Rafels-Ybern et al., 2018; Rafels-Ybern et al., 2019). While some bacterial taxa have expanded their repertoire of encoded A34 tRNAs, other bacteria have lost the inosine modification and modifying enzyme completely (Yokobori et al., 2013; Rafels-Ybern et al., 2018; Rafels-Ybern et al., 2019).

Until the 2018 genomic analysis study, unmodified A34 tRNAs had only been observed in organisms and organelles with highly reduced genomes or studied as isolated or prepared mutant tRNAs. The translation of organellar and *Mycoplasma* genomes employ greatly reduced tRNA complements relative to complements in typical bacteria and eukaryotic systems. The *Mycoplasma* and organellar-like interactions have been referred to as “super-wobble” interactions (Rogalski et al., 2008). Yeast mitochondria encode a single unmodified arginine tRNA with an ACG anticodon as the only tRNA capable of decoding the CGN codon box. Unlike bacteria, yeast mitochondria rarely use CGN codons to encode arginine. The function of this tRNA species has not been experimentally evaluated. *Mycoplasma* species employ an authentic A34 threonine tRNA (Andachi et al., 1987; Samuelsson et al., 1987). The function of this tRNA has been evaluated in *in vitro* translation experiments employing *Mycoplasma* S30 extracts. These experiments show that the A34 threonine tRNA is capable of decoding ACU, ACC, and ACG codons (Inagaki et al., 1995).

The function of A34 tRNAs have been evaluated in the context of glycine and serine tRNA backgrounds in *E. coli*-derived *in vitro* translation systems. An overexpressed and purified *E. coli* glycyl tRNA_ACC_ strongly decoded GGU, weakly decoded GGC, and surprisingly competed with glycyl tRNA_UCC_ to decode both GGA and GGG (Boren et al., 1993). A closely-related *in vitro* translation experiment examining the properties of A34 tRNAs in the context of a Ser tRNA backbone showed that A34 anticodons strongly discriminated and read predominantly U-ending codons, with minor reading of C-ending codons and no apparent reading of A- or G-ending codons in the Ser UCN codon box (Takai et al., 1999).

For 10 of the 15 codons evaluated, authentic orthogonal A34 tRNAs show a strong preference for U3 as opposed to C3 codons. Discrimination ratios are greater than or equal to approximately 10 to one for *M. jannaschii* A34 tRNAs targeting codons for Phe, Cys, Ile, Thr, Asn, and Ser (both possible A34 anticodons). Orthogonal A34 tRNAs for Leu and His exhibit high discrimination ratios in variants that are not inosine modified. No orthogonal tRNA with an ACG anticodon, targeting the Arg CGU codon, that was not predominantly inosine modified was identified. Generation of an orthogonal tRNA_ACG_ with an authentic A34 is of particular interest as it would allow characterization of the competition between orthogonal tRNA_ACG_ and endogenous tRNA_ICG_.

The codons for proline, alanine, and glycine include GC pairs in the first two positions of the codon. That the third position of interaction in codon/anticodon recognition is less important as a result of the strength of the G/C interactions describes the expectation of the “two-out-of-three” and “strong and weak” codon hypotheses (Lagerkvist, 1978; Grosjean and Westhof, 2016). The low U3:C3 discrimination ratios as well as detectable decoding of the G-ending codons for each of these authentic A34 orthogonal tRNAs support these interpretations of genetic code reading. In the cases of proline and alanine, the A34 tRNAs essentially do not discriminate between C- and U-ending codons. In the case of glycine, the U-ending codon is slightly preferred. The arginine four box is the fourth codon box with 2 GC pairs at the first two codon positions. As mentioned, a non-inosine modified version of this tRNA anticodon could not be identified.

## 5 Conclusion

We set out to explore the extent to which *M. jannaschii* tyrosyl tRNAs with ANN anticodons were modified to inosine by *E. coli* TadA. Given that apparent quantitative A to I modification was observed in the orthogonal *M. jannaschii* tRNA_ACG_, which possesses an anticodon identical to the only *E. coli* tRNA species that undergoes A to I modification (tRNA^Arg2^), and that the closely-related orthogonal tRNA_AUG_ was highly modified, we hypothesized that orthogonal *M. jannaschii* tRNAs with anticodons similar to ACG (i.e., ANG or ACN) may also be substrates for *E. coli* TadA. We also recognized that the heterodimeric ADATs which are responsible for modifying 8 A34 tRNAs to inosine in eukaryotes evolved from the bacterial TadA, and that *E. coli* TadA could have broader substrate recognition than had been previously observed. We expected the degree to which an orthogonal A34 tRNA was able to distinguish between U- and C-ending codons would correlate with the extent of A-to-I modification, as unmodified A34 tRNAs are expected to have a strong preference for pairing with U in the third codon position.

The expectation that A34 tRNAs show a strong preference for U-ending codons was based on a limited set of sometimes conflicting experimental data on the function of A34 tRNAs in translation as well as our previous observation that “anomalous” decoding of histidine CAC codons by the *M. jannaschii* tRNA_AUG_ was due to inosine modification (Boren et al., 1993; Inagaki et al., 1995; Takai et al., 1999; Chen et al., 2002; Biddle et al., 2016; Agris et al., 2018). Anticodon loop sequence variants of tRNA_AUG_ that abrogated inosine modification displayed high level discrimination between U- and C-ending codons. We anticipated that U3:C3 discrimination ratios might provide insight on inosine modification states of corresponding A34 tRNAs.

We observed a wide range of U3:C3 discrimination ratios, yet sequencing indicated inosine modification in only three out of fifteen tRNAs evaluated. Thus, orthogonal pair directed sense codon reassignment using the *M. jannaschii* tRNA/aaRS pair enabled a wider examination of the function of authentic A34 tRNAs in *in vivo* translation than has previously been performed. For the most part, unmodified A34 tRNAs largely pair with only U3 codons as the original wobble rules suggest. In the instances with GC pairs in the first two positions of the codon (i.e., proline, alanine, and glycine), unmodified A34 tRNAs readily decode the corresponding C- and G-ending codons. In only one instance is decoding of an A-ending codon by an authentic A34 tRNA observed. tRNA_AGG_ decoding each codon in the proline four box may represent a unique situation pertaining to proline as opposed to an indication of broader A34/A3 pairing tendencies. Inosine modification explains the other A-ending codon reassigned with an efficiency above the limit of detection.

This evaluation demonstrates that authentic A34 tRNAs perform effectively in the context of the *E. coli* translation apparatus (Chen et al., 2002). Our data offer no apparent functional reason why A34 tRNAs did not evolve to translate codon four boxes. The absence of unmodified A34 tRNAs across the domains of life suggests that more than just pressure for reduced tRNA complements is responsible for the absence of A34 tRNAs, but a clear functional deficiency is not evident in *E. coli*.

## Data Availability

The raw data supporting the conclusion of this article will be made available by the authors, without undue reservation.
