# Supplementary material for: Codon decoding by orthogonal tRNAs interrogates the in vivo preferences of unmodified adenosine in the wobble position
Source: Front Genet. 2024 Apr 19;15:1386299. doi: 10.3389/fgene.2024.1386299 (PMC11066159; doi:10.3389/fgene.2024.1386299)
Supplement: Supplementary file 1 [file DataSheet1.PDF]

Supplementary Material

S.1. Cell strains

NEB 5-alpha (New England Biolabs): *fhuA2 Δ(argF-lacZ)U169 phoA glnV44 Φ80 Δ(lacZ)M15 gyrA96 recA1 relA1 endA1 thi-1 hsdR17*

SB3930 (Yale CGSC): *λ-*, *ΔhisB463*

S.2. General materials and reagents

All restriction enzymes, DNA polymerases, and T4 kinase were purchased from New England Biolabs and used according to the manufacturer's instructions. ATP was purchased from Fisher (BP413-25) and dNTPs were purchased from New England Biolabs (N0447S). DNA isolation was performed using a Thermo Scientific GeneJET plasmid miniprep kit (K0503) according to the manufacturer's protocols. Intermediate cloning steps and PCR products were purified using a Thermo Scientific GeneJET PCR spin kit (K0701).

LB liquid media (per liter: 10 g tryptone, 5 g yeast extract, 5 g NaCl) and LB agar plates with 15 g/L agar were used unless otherwise noted. Isopropyl-beta-D-thiogalactoside (IPTG) was purchased from Gold Bio (I2481C5). Spectinomycin (Enzo Life Science, BML-A281) was used at 50 μg/mL to maintain the vectors harboring the tRNA and aaRS genes. Carbenicillin (PlantMedia, 40310000-2) was used at 50 μg/mL to maintain the vectors harboring the GFP reporter gene. All bacterial cultures were grown at 37 °C unless otherwise noted. All liquid cultures were shaken at 225 rpm unless otherwise noted.

Electrocompetent stocks of all strains were prepared in-house according to the method of Sambrook and Russell (J. Sambrook and D. W. Russell *Molecular cloning: a laboratory manual*. **2001**, Cold Spring Harbor Laboratory press). Typical transformation efficiencies for electrocompetent cells produced in this way are 10^9 cfu/μg of supercoiled DNA. All transformations were recovered in SOC (20 g/L tryptone, 5 g/L yeast extract, 10 mM NaCl, 2.5 mM KCl, 10 mM $MgCl_2$, 20 mM glucose) for 1 hour at 37 °C with shaking prior to transfer to media containing appropriate antibiotics and/or inducers as noted.

All oligonucleotides were purchased from Integrated DNA Technologies (Coralville, Iowa, USA). DNA sequencing was performed by Azenta/Genewiz (Plainfield, NJ, USA).

Supplementary Table 1. Oligonucleotide primers for reverse transcription, amplification, and sequencing of *M. jannaschii* tRNAs

Primer name	Sequence	Primer use
Mj-RevTrans	GAC ACG GTA CCA CAC AAC TGG GCA ACG CAA CCT AGC TAA TGG TCC GGC GGA GGG GAT TTG	Reverse transcription of *M. jannaschii* tRNA
cDNA-amp-fwd	GAC ACG GTA CCA CAC AAC TGG	Amplification of cDNA from *M. jannaschii* tRNA<br>Sequencing of PCR products
cDNA-amp-rev	GGC GTA TCT GCG CAG TAA GAT GCG CCC CGC ATT CCG GCG GTA GTT CAG CAG GGC AGA ACG	Amplification of cDNA from *M. jannaschii* tRNA

Supplementary Table 2. Number of biological replicates analyzed for each orthogonal anticodon/GFP reporter codon pair

tRNA Anticodon	Codon targeted	PQU	PQC	PQA	PQG
**AAA**	Phe UUU	11	6	-----	-----
**AAG**	Leu CUU	27	21	12	6
**AAU**	Ile AUU	21	15	6	6
**AAC**	Val GUU	12	6	6	6
**AGA**	Ser UCU	21	15	6	6
**AGG**	Pro CCU	31	27	18	6
**AGU**	Thr ACU	15	15	6	6
**AGC**	Ala GCU	18	11	6	6
**AUG**	His CAU	12	12	6	-----
**AUU**	Asn AAU	12	12	6	6
**AUC**	Asp GAU	18	12	12	12
**ACA**	Cys UGU	27	15	-----	-----
**ACU**	Arg CGU	12	12	12	12
**ACU**	Ser AGU	35	18	12	12
**ACC**	Gly GGU	18	12	6	6

Values highlighted in pale rose indicate systems for which codon reassignment efficiencies are newly reported in this paper.

S.3 GFP reporter vectors for codon reassignment

Oligonucleotide primers for construction of each GFP reporter codon variant has been described, even if the codon reassignment efficiency for a particular combination of orthogonal tRNA anticodon and GFP reporter codon is previously unreported. The full vector sequence for the parent GFP reporter has also been reported.

1) W. Biddle, M. A. Schmitt, and J. D. Fisk, Evaluating Sense Codon Reassignment with a Simple Fluorescence Screen, *Biochemistry*, **2015**, *54*, 7355-7364.
2) M. A. Schmitt, W. Biddle, and J. D. Fisk, Mapping the Plasticity of the *Escherichia coli* Genetic Code with Orthogonal Pair-Directed Sense Codon Reassignment. *Biochemistry*, **2018**, *57*, 2762-2774.
3) D. G. Schwark, M. A. Schmitt, W. Biddle, and J. D. Fisk, The Influence of Competing tRNA Abundance on Translation: Quantifying the Efficiency of Sense Codon Reassignment at Rarely Used Codons, *ChemBioChem*, **2020**, *21*, 2274-2286.

S.4 Orthogonal translation machinery vectors

The sequence of the full vector backbone from which the *M. jannaschii* tRNA/aaRS variants are expressed for evaluation using the fluorescence-based screen was reported in W. Biddle, M. A. Schmitt, and J. D. Fisk, Evaluating sense codon reassignment with a simple fluorescence screen, *Biochemistry*, **2015**, *54*, 7355-7364. No changes to the vector were made beyond varying the anticodon.

The gene sequence for the *M. jannaschii* tRNA variants ("nnn" is the anticodon, positions 34-36) is:

```
5′ - CCGGCGGTAGTTCAGCAGGGCAGAACGGCGGACTnnnAATCCGCATGGCAGGGGTTCAAAT
CCCCTCCGCCGGACCA - 3′
```

Each tRNA anticodon variant discussed as part of this study has been evaluated for reassignment of at least one codon previously, save the variant to reassign the Arg CGU codon (anticodon ACG). Sequences of the mutagenic oligonucleotides used to generate each anticodon variant are included as supplementary material within the manuscript in which they are originally described.

The sequence of the oligonucleotide primer used to generate *M. jannaschii* $tRNA_{ACG}$ is:

5′ - GCA GAA CGG CGG ACT **ACG** AAT CCG CAT GGC AGG GGT TC - 3′

S.5 Fluorescence-based screen for codon reassignment and calculation of reassignment efficiency

Superfolder green fluorescent protein (GFP) reporter plasmids were co-transformed with vectors expressing the modified orthogonal translational components into *E. coli* SB3930. After overnight growth, colonies were picked into 200 µL LB media in a 96 well plate. Cells were grown to at least mid-log phase (typically 6-8 hours) with shaking at 37 °C. Cells were diluted 10-fold into LB media with antibiotics to maintain the plasmids and 1 mM IPTG for induction of both the aaRS and GFP. Assays were performed in a Fluorotrac 200 clear bottom 96 well plate (Greiner 655096) and monitored in a BioTek Synergy H1 or BioTek Synergy Neo 2S plate reader at 37 °C with continuous double orbital shaking. The optical density (OD600) and fluorescence of each well was measured every 15

minutes for at least 15 hours; optical density was measured at 600 nm, and fluorescence was measured with an excitation at 485 nm and detection at 515 nm with a 20 nm band pass.

For each biological replicate, the relative fluorescence (corrected fluorescence per OD) was calculated for each of the 16 data points gathered between 8 and 12 hours after induction of GFP and the aaRS with IPTG. The 16 relative fluorescence values were averaged to determine the RFU for each sample. That RFU is divided by the average RFU for all biological replicates of the 100% reassigning fluorescence control (wild type GFP) to determine the reassignment efficiency. Sense codon reassignment efficiency for each tRNA anticodon/GFP codon variant pair was calculated by averaging the reassignment efficiency for at least six biological replicates. The number of biological replicates used to calculate each reassignment efficiency reported here is shown in Supplementary Table 2.

An extremely detailed discussion and workflow for calculating codon reassignment efficiency was provided in the Supporting Information for M. A. Schmitt, W. Biddle, and J. D. Fisk, Mapping the Plasticity of the *Escherichia coli* Genetic Code with Orthogonal Pair-Directed Sense Codon Reassignment. *Biochemistry*, **2018**, *57(19)*, 2762-2774.

Supplementary Figure 1:

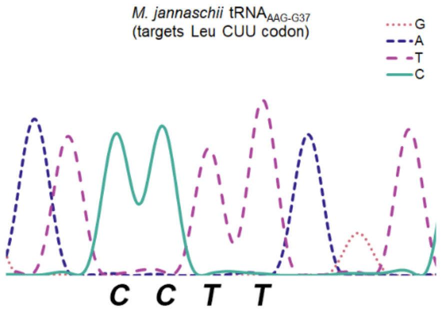

Reassignment efficiency of tRNA$_{AAG-G37}$:

Leu CUU: 5.4 ± 0.08%

Leu CUC: 0.8 ± 0.04%

U3:C3 discrimination: 87:13

Supplementary Figure 1. Chromatogram trace of the A37G anticodon loop variant of tRNA$_{AAG}$ (targets Leu CUU). Cytosine is incorporated in response to G37. Only thymidine is incorporated in response to the nucleotide at position 34. No deamidation of adenosine 34 to inosine is detected; this anticodon loop variant is no longer able to be recognized by *E. coli* TadA. These reassignment efficiencies are newly reported.

Supplementary Figure 2:

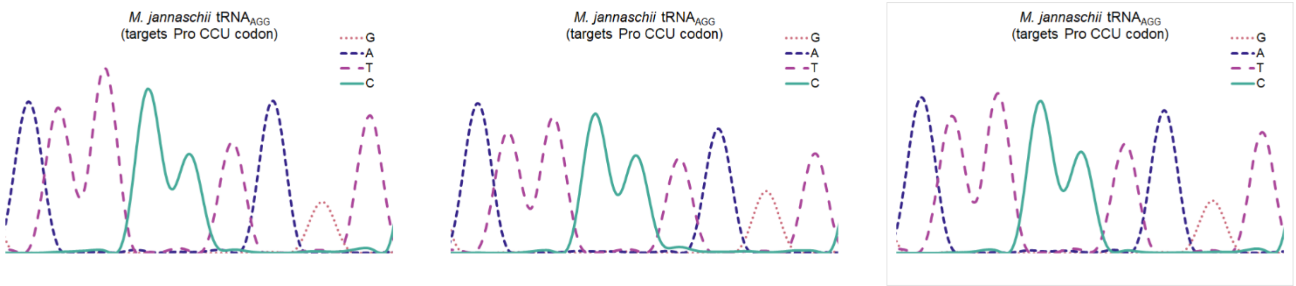

Supplementary Figure 2. Chromatogram traces of additional independent preparations of reverse transcribed orthogonal tRNAs with an AGG anticodon targeted to reassign proline CCU codons. The absence of detectable inosine modification was surprising given the orthogonal tRNA's ability to decode both the CCU and CCC codons with nearly equal efficiency coupled with at least partial inosine modification detected for each of the other orthogonal tRNAs with an ANG anticodon. A very small amount of signal for cytosine is detected in one of the four traces (three here, one in the main text). However, we do not believe it is indicative of inosine modification as a) the bump is not reproducible across different preparations and b) the intensity is similar to that of the noise observed at other nucleotide positions across the collected sequences for all of the orthogonal A34 tRNAs.
